# Sex-specific patterns of senescence in artificial insect populations varying in sex-ratio to manipulate reproductive effort

**DOI:** 10.1186/s12862-020-1586-x

**Published:** 2020-02-03

**Authors:** Charly Jehan, Manon Chogne, Thierry Rigaud, Yannick Moret

**Affiliations:** UMR CNRS 6282 BioGéoSciences, Équipe Écologie Évolutive, Université Bourgogne-Franche Comté, Dijon, France

**Keywords:** Ageing, Cost of reproduction, Disposable soma theory, Immunity, Immuno-senescence, *Tenebrio molitor*

## Abstract

**Background:**

The disposable soma theory of ageing assumes that organisms optimally trade-off limited resources between reproduction and longevity to maximize fitness. Early reproduction should especially trade-off against late reproduction and longevity because of reduced investment into somatic protection, including immunity. Moreover, as optimal reproductive strategies of males and females differ, sexually dimorphic patterns of senescence may evolve. In particular, as males gain fitness through mating success, sexual competition should be a major factor accelerating male senescence. In a single experiment, we examined these possibilities by establishing artificial populations of the mealworm beetle, *Tenebrio molitor*, in which we manipulated the sex-ratio to generate variable levels of investment into reproductive effort and sexual competition in males and females.

**Results:**

As predicted, variation in sex-ratio affected male and female reproductive efforts, with contrasted sex-specific trade-offs between lifetime reproduction, survival and immunity. High effort of reproduction accelerated mortality in females, without affecting immunity, but high early reproductive success was observed only in balanced sex-ratio condition. Male reproduction was costly on longevity and immunity, mainly because of their investment into copulations rather than in sexual competition.

**Conclusions:**

Our results suggest that *T. molitor* males, like females, maximize fitness through enhanced longevity, partly explaining their comparable longevity.

## Background

Life history theory assumes that organisms are constrained to optimally trade-off limited energetic and time resources between reproduction and life span, to maximize fitness [1, 2]. This principle is at the core of the theory of ageing, which predicts that, as reproduction is resource demanding, current reproduction is traded-off against future reproduction and survival, caused by a reduced investment into somatic protection and maintenance [2–4]. However, recent studies have sometimes revealed patterns of actuarial (decline in survival rate with age) and reproductive (decline in reproductive success with age) senescence rather contrasted with this prediction [5–7]. Since individuals may differ in both resource acquisition and resource allocation between traits, depending on individual and environmental quality, the cost of reproduction can remain undetected at the population level [8, 9].

Studies that investigated cost of reproduction in terms of senescence mainly focused on females [10, 11]. Those on males often referred to sexual selection theory and therefore on the cost of producing and maintaining sexual traits [12]. In males, cost of reproduction may result from resource demands for courtship, mating, struggling with female resistance, mate guarding, the production of sperm and accessory gland proteins [13–16]. They may also engage into costly intra-sexual competition for females through pre- and post-copulatory contests with other males [17]. In females, cost of reproduction may result from gamete production, offspring care, harassment by males, mating injuries, sexually transmitted diseases and damaging seminal substances [18–21]. These differential costs may have contributed to the evolution of sexually dimorphic life-history strategies in many species through which males and females achieve maximal fitness. For instance, while males may maximize fitness by increasing mating success at the expense of longevity, females may maximize fitness through longevity because offspring production, although resource intensive, requires time too. The different reproductive costs may also contribute to different patterns of senescence between males and females, which may vary within and among populations, depending on their relative intensity. Strong investment into reproduction early in life seems to contribute to accelerating reproductive and actuarial senescence [22]. However, our understanding of the impacts of the costs of reproduction on senescence mainly relies on theoretical and correlative studies, whereas experimental investigations are still scarce.

Somatic protection partly depends on the immune system, whose competence may diminish with age. Such an immunosenescence causes enhanced sensitivity to infection and inflammatory diseases, increasing risk of morbidity and mortality with age [23, 24]. Increased reproductive effort was found associated with enhanced susceptibility to parasitism and disease [25] or decreased immune activity [17, 26–29]. Trade-offs between reproductive and immune functions for limited resources, or negative pleiotropic effects of reproductive hormones on immune defence, have been proposed as proximate causes of the cost of reproduction [30]. However, contradictory results are common as studies also failed to demonstrate such a cost [31–33]. If investment into reproduction can induce a progressive decline in somatic functions, strong investment into early reproductive effort may generate accelerated immunosenescence and contribute to actuarial senescence.

Recent correlative evidence suggests that population structure, such as sex-ratio, affects individual reproductive effort with potential sex-specific consequences on senescence [34, 35]. In particular, variation in sex-ratio is predicted to modulate the cost of mating, through the strength of sexual selection in males [36], influencing the putative trade-off between reproductive effort and somatic maintenance [11]. Furthermore, cost of reproduction in females is also predicted to depend on population sex-ratio as it is expected to influence male competition for fertilization [16]. Hence, experimentally varying population sex-ratio appears to be a valuable tool to manipulate males and females reproductive effort and test its impacts on senescence at the population level.

Here, in a single experiment, we investigated the consequences of variable levels of investment in breeding effort on lifetime reproduction, survival and immunity of males and females of the mealworm beetle, *Tenebrio molitor*, of which we have manipulated the sex-ratio in artificial populations. In this highly promiscuous insect, manipulating the sex-ratio of populations is expected to affect both the average intensity of intra sexual competition or sexual selection, and individual mating rate. In male-biased sex-ratio conditions, males should face fewer mating opportunities, whereas females should show high individual reproductive effort. By contrast, in female-biased sex-ratio condition, males should copulate more frequently, whereas females should have fewer opportunities to mate. This experimental design allowed us to test the cost of different key features of male and female reproduction in terms of senescence at the population level by examining their lifetime changes in survival, fertility, reproductive effort, body condition and immunity. Note, however, that manipulating sex-ratio may only affect the *opportunity* for sexual selection and not the *actual* sexual selection [37], and life-history particularities of biological models should be taken into account. For example, common wisdom is that male-biased sex-ratio conditions should accelerate male reduction of survival, reproduction and immunity because of intense pre- and post-copulatory intra sexual competition. However, in *T. molitor*, mating might be where the largest costs arise in reproduction for males (see below), and accelerated senescence is expected in populations with female-biased sex-ratio because males should produce higher reproductive effort. Indeed, direct observations of the mating behaviour of *T. molitor* suggest that males do not engage in costly physical contests to access females [38, 39]. Courtship and mating are relatively brief during which males transfer a spermatophore that does not release the sperm before 7–10 min post-copulation [40]. Males may then perform rather passive short post-copulatory mate guarding, consisting on staying within 1 cm of the female for more than.

One minute in the presence of competitors [38, 39]. However, males do not appear to have evolved specific post-copulatory mate-guarding behaviour like those observed in other insects [41]. The spermatophore transferred during copulation contains nutrient-rich substances that constitute a nuptial gift [42], whose cost may prevent males to copulate again for 20 min after the last copulation [41]. Hence, as mating is more costly than pre- and post-copulatory sexual competition, *T. molitor* males may best achieve fitness through longevity, just like females, which would ultimately prevent the evolution of divergent patterns of actuarial senescence between males and females. Females, for their part, may exhibit strong early reproductive effort in populations with male-biased sex-ratio, they also should exhibit accelerated decline in reproduction, and immunity or earlier immune dysregulation, correlating with reduced survival with age. In populations with female-biased sex-ratio, females should survive, reproduce and maintain immunity at older age, as they might exhibit lower early life reproductive effort.

## Methods

### Mealworm beetles

Mealworm beetles are stored grain product pests that live several months in populations of variable density and at sex-ratio of about 50% (± 20%). *T. molitor* males and females may initiate reproduction from the fifth day post emergence, although they reach their full sexual maturity from the eighth day post emergence. They can mate many times with several partners within their 2 to 5 months of adult life. Females are continuously receptive to mating during adulthood and may produce up to 30 eggs per day although egg production may decline after 3 weeks [43]. Although able to store sperm in their spermatheca, females need to mate frequently to maintain high egg production [44].

The immune system of *T. molitor* relies on both constitutive cellular (e.g. haemocytes) and enzymatic (e.g. prophenoloxidase system) components at the core of the inflammatory response [45]. Their activity is cytotoxic [46], causing self-damage [47] and lifespan reduction [48–51]. They were found to decrease after mating [52] and either decline [53] or increase [54] with age. In addition, the inducible production of antibacterial peptides in the haemolymph [45] is an energetically costly process that may reduce survival [55]. As selection on immune expression and immune regulation might be weaker after reproductive senescence, age-related decline of baseline levels of immunity might be observed and immune activation may occur at old age due to dysregulation [54, 56].

### Artificial populations and experimental design

Virgin adult beetles of controlled age (10 ± 2 days post-emergence) were obtained from pupae haphazardly sampled from a stock culture maintained in laboratory conditions (24 ± 2 °C, 70% RH in permanent darkness) at Dijon, France. Prior to the experiments, all these experimental insects were maintained separately in laboratory conditions, and supplied ad libitum with bran flour and water, supplemented by apple.

Fifteen artificial populations of 100 adult beetles were made according to three sex-ratio conditions. Five populations had a balanced sex-ratio, each comprising 50 males and 50 females (thereafter named the 50%_males condition), and were considered as the reference populations. Five populations had a male-biased sex-ratio, each comprising 75 males and 25 females (75%_males). Finally, five populations had a female-biased sex-ratio, each comprising 25 males and 75 females (25%_males). Each population was maintained in a plastic tank (L × 1 x H, 27 × 16.5 × 11.5 cm) containing bran flour, supplied once a week with apple and water. Every 2 weeks, each population was transferred into a clean tank supplied with fresh bran flour, thus avoiding the development of the progeny with the experimental adults.

### Age specific reproductive assay

Reproductive capacity of females and males in each population was estimated weekly. To this purpose, 4 females haphazardly picked in each population were individually transferred into a plastic Petri dish (9 cm in diameter), containing bleached flour, a 2 mL centrifuge tube of water and a piece of apple. Each female was allowed to lay eggs in the Petri dish for 3 days, and was then returned to their initial population box. Two weeks later, the number of larvae was counted in each Petri dish to quantify female fertility, which is the number of viable larvae produced per female [57].

Concomitantly, four males haphazardly picked in each population were also individually transferred into Petri dishes, as above. Reproductive success of males was estimated through direct measures of their fertility (number of viable offspring per male [57]) instead of measuring spermatophores or counting the sperm, which are rough surrogates of male reproductive success. Each male was provided with a virgin female aged from 8 to 15 days for 24 h and was then returned in its initial population. Each female was then allowed to lay eggs in the Petri dish for three additional days to estimate, as described above, male fertility. In *T. molitor*, males may affect female fecundity (number of potential eggs produced by the female) and therefore their fertility, according to the respective quality of spermatophores and sperm transferred during mating. Consequently, male’s success was a measure of the *potential* reproductive effort, not the one realized within its experimental population.

While assayed for their reproduction, focal females and males were replaced by marked individuals of the same age and sex in all the populations, to keep sex-ratio and density constant. Substitutes were from the same cohort as the experimental insects, kept in a separate tank of mixed-sex population. They were marked by clipping a piece of one elytra. When focal insects assayed for their reproduction were returned into their initial population box, substitutes were removed and returned into their tank.

### Estimation of male and female reproductive effort at the population level

Survival of the insects was checked weekly, and dead insects were replaced by marked substitutes of the same sex and about the same age to keep the population sex-ratio and density of individuals constant. No measurement was performed on these marked individuals.

As the experimental design does not allow gathering measurements of longevity and fertility for each individual of the population, we estimated male and female reproductive effort (RE) at the population level, from the above age-specific measures of fertility, for each of the five population replicates, within sex-ratio conditions. This estimate was calculated as the total number of viable larvae produced per female or male in each replicate (i.e. the cumulative number of larvae produced during the whole experiment in a given replicate divided by the number of females or males tested for this replicate), divided by their respective average lifespan in the population replicate (i.e. the average lifetime of females or males in each relicate). The equation is given as follow:

$$ {RE}_r=\frac{l}{ML} $$

Where *l* is the total number of offspring (here viable larvae) produced per assayed females or males in the population replicate *r*, and *ML* is the recorded mean lifespan (in weeks) of males and females in the replicate *r*. RE values (as offspring per individuals and per mean weeks of survival in the population) of each sex and population replicate within each sex-ratio condition were used as data points for comparisons between modalities of sex-ratio.

Note that female RE values are likely representative of both female and male conditions resulting from the experiment, because the female reproductive performance resulted from mating with males from their respective population. By contrast, male RE values are representative of the male condition only, because male reproductive performance was standardized by pairing it with a virgin and age-controlled female that did not experience the experimental conditions. Therefore, male RE must be seen as a surrogate of male reproduction potential.

### Body condition and haemolymph collection

At weeks 2, 4, 6 and 12 after the start of the experiment, 4 females and 4 males were picked at random in each population to estimate their body condition and immunity. The first three time points were chosen as being relevant of the time period during which most of the beetle reproduction is achieved and survival is still relatively high [44]. It is also within this period of time that a potential decline in somatic protection, including immunity, is predicted. The last time point corresponds to a period of time when reproduction should have almost ceased and when few beetles remain alive. As immunity measurements was destructive sampling, sampled insects were replaced by marked substitutes as above, to keep sex-ratio and density constant. However, this substitution was definitive, as sampled individuals were not returned to their initial population box after being assayed. The below estimation of the insect body condition and immunity was done as described in [58]. Beetles were first sized by measuring the length of the right elytra with Mitutoyo digital callipers (precision 0.1 mm) and weighed to the nearest mg with an OHAUS balance (discovery series, DU114C). Body condition was then estimated by the residuals of the regression between body size and body mass. Then, beetles were chilled on ice for 10 min before the sampling of 5 μL of haemolymph from a wound made in the beetle’s neck and flushed in a microcentrifuge tube containing 25 μL of phosphate-buffered saline (PBS 10 mM, pH 7.4). A 10-μL aliquot was immediately used to measure haemocyte count. Another 5-μL aliquot was kept in an N-phenylthiourea-coated microcentrifuge tube (P7629, Sigma-Aldrich, St Louis, MO, USA) and stored at − 80 °C for later examination of its antibacterial activity. The remaining haemolymph solution (15 μL) was further diluted in 15 μL of PBS and stored at − 80 °C for later measurement of its phenoloxidase activity.

### Immune parameters

Haemocyte count was measured using a Neubauer improved haemocytometer under a phase-contrast microscope (magnification × 400).

Antimicrobial activity of the haemolymph was measured using the inhibition zone assay described in [58]. Briefly, an overnight culture of the bacterium *Arthrobacter globiformis* from the Pasteur Institute (CIP105365) was added to a Broth medium containing 1% agar to achieve a final concentration of 10^5^ cells.mL^− 1^. Six millilitres of the medium was subsequently poured per Petri dish and, after solidification, 12 wells were made inside the agar plate in which 2 μL of each haemolymph sample was deposited. Plates were then incubated overnight at 28 °C and the diameter of each zone of inhibition was measured.

For each haemolymph sample, both (i) the activity of naturally activated phenoloxidase (PO) enzyme only (hereafter PO activity) and (ii) the activity of PO plus that of proenzymes (proPO) (hereafter Total-PO activity) were measured using the spectrophotometric assay described in [59]. Total-PO activity quantification required the activation of proPO into PO with chymotrypsin, whereas PO activity was measured directly from the sample. Frozen haemolymph samples were thawed on ice and centrifuged (3500 g, 5 min, 4 °C). In a 96-well plate, 5 μL of supernatant were diluted in 20 μL of PBS and were added either 140 μL of distilled water to measure PO activity or 140 μL of 0.07 mg. mL^− 1^ chymotrypsin solution (Sigma-Aldrich, St Louis, MO, USA, C-7762) to measure Total-PO activity. Subsequently, 20 μL of a 4 mg.mL^− 1^ L-Dopa solution (Sigma-Aldrich, St Louis, MO, USA, D-9628) were added to each well. The reaction proceeded for 40 min at 30 °C, in a microplate reader (Versamax, Molecular Devices, Sunnyval, CA, USA). Reads were taken every 15 s at 490 nm and analysed using the software SOFT-Max®Pro 4.0 (Molecular Devices, Sunnyvale, CA, USA). Enzymatic activity was measured as the slope (Vmax value: change in absorbance unit per min) of the reaction curve during the linear phase of the reaction and reported to the activity of 1 μL of pure haemolymph.

### Statistics

Cox-regressions with a time-dependent covariate were used to analyse the difference in survival rates with respect to sex-ratio during the time (in weeks) from the start of the experiment and the death of all individuals. Sex-ratio was coded as categorical variables. The effect of sex ratio in the statistical model used the reference survival function generated from the data derived from the females or the males of the 50%-male sex-ratio condition. Time (in weeks) was incremented as a covariate in interaction with sex-ratio in the model as hazard ratios when the survival functions where not constant over time (for more details, see [60]). The analyses of fertility (i.e. the number of larvae produced per female or male at each week) and immune parameters were performed using mixed models, either Linear or Generalized linear depending on the nature of the data (see table legends). Starting models included sex-ratio condition, week (continuous variable for fertility, ordinal variable for immunity), their interaction, body condition and replicates treated as a random factor (REML estimates of variance component). The models presented here are those minimizing the AICc, where ΔAICc > 2 is usually considered to be good support [61], after comparisons of all models including predictors and their interactions, in a stepwise fashion (see Additional file 1: Table S1). The analyses of reproductive effort were made using ANOVA testing the effect of sex-ratio conditions. Analyses were made using IBM® SPSS® Statistics 19, JMP v. 10.0 and R version 3.3.2 (The R Foundation for Statistical Computing, Vienna, Austria, http://www.r-project.org). All the data files are available from the Dryad data base [62].

## Results

### Demography: survival, fertility and reproductive effort

**Fig. 1:**
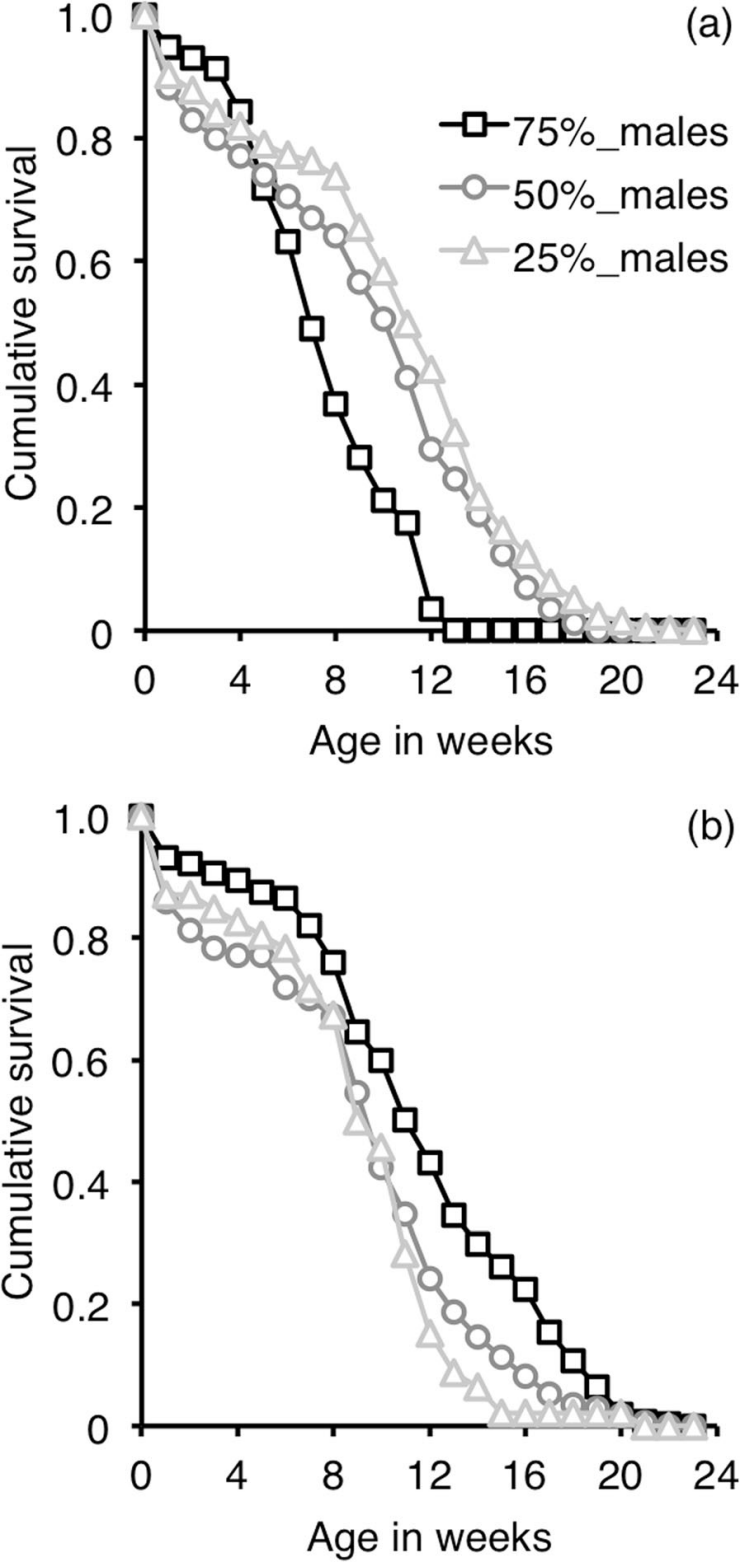
Age-specific survival according to sex-ratio condition. **a** females; **b** males

A first survival analysis comparing males and females of the 50%-male sex-ratio condition, which presumably corresponds to the sex-ratio condition in natural populations of *T. molitor*, showed no difference in longevity between males and females (Cox regression: Wald statistics = 0.004, d.f. = 1, *p* = 0.947, see Additional file 1: Figure S1). Survival of females and males was significantly affected by the sex-ratio condition (Table 1, Fig. 1). In the 75%-male sex-ratio condition, females exhibited an accelerated mortality with time by a factor of 13% per week compared to females of the 25 and 50%-male sex-ratio conditions (see odd ratio of Sex-ratio*Time-Cov in Table 1a). There was no significant difference in survival between females in the 25 and 50%-male sex-ratio conditions (Table 1a, Fig. 1a). Contrasting with females, males in the 75%-male sex-ratio condition survived significantly longer than those in the 25 and 50%-male sex-ratio conditions (by 53 and 50%, respectively, see odd ratio in Table 1b, Fig. 1b).

**Table 1:** Survival of adult females (a) and males (b) of *Tenebrio molitor* according to sex-ratio condition (Sex-ratio). The “simple” contrast was used for Sex-ratio (survival of males in the 50% of male condition was used as baseline). For females (a), a time-dependent procedure was used to account for the time-dependent effect of Sex-ratio on the risk of mortality (*T* × Sex-ratio). This procedure was not necessary for males as the effect of Sex-ratio on the risk of mortality was constant over time (b)

A. Variables	*b*^a^	*s.e.*^b^	*Wald*^c^	*d.f.*	*p*^d^	*Odds ratio*^e^
Sex-ratio			1.61	2	0.744	
*T* × Sex-ratio			9.77	2	**0.008**	
75% of males vs 50% of males	0.12	0.04	9.28	1	**0.002**	1.13
25% of males vs 50% of males	0.00	0.02	0.05	1	0.831	1.00
75% of males vs 25% of males	0.12	0.04	9.03	1	**0.003**	1.12
B. Variables	*b*	*s.e.*	*Wald*	*d.f.*	*p*	*Odds ratio*
Sex-ratio			21.34	2	**< 0.001**	
75% of males vs 50% of males	−0.38	0.10	15.36	1	**< 0.001**	0.70
25% of males vs 50% of males	0.15	0.17	0.82	1	0.366	1.16
75% of males vs 25% of males	−0.53	0.16	10.98	1	**0.001**	0.59

^a^*b* = regression coefficient of overall survival function for variables

^b^Standard error of regression coefficient

^c^Wald statistic for variable

^d^Significant level for Wald statistic. Values *p* ≤ 0.005 are given in bold

^e^Odds ratio of survival for variable relative to control (=exp.(b))

**Fig. 2:**
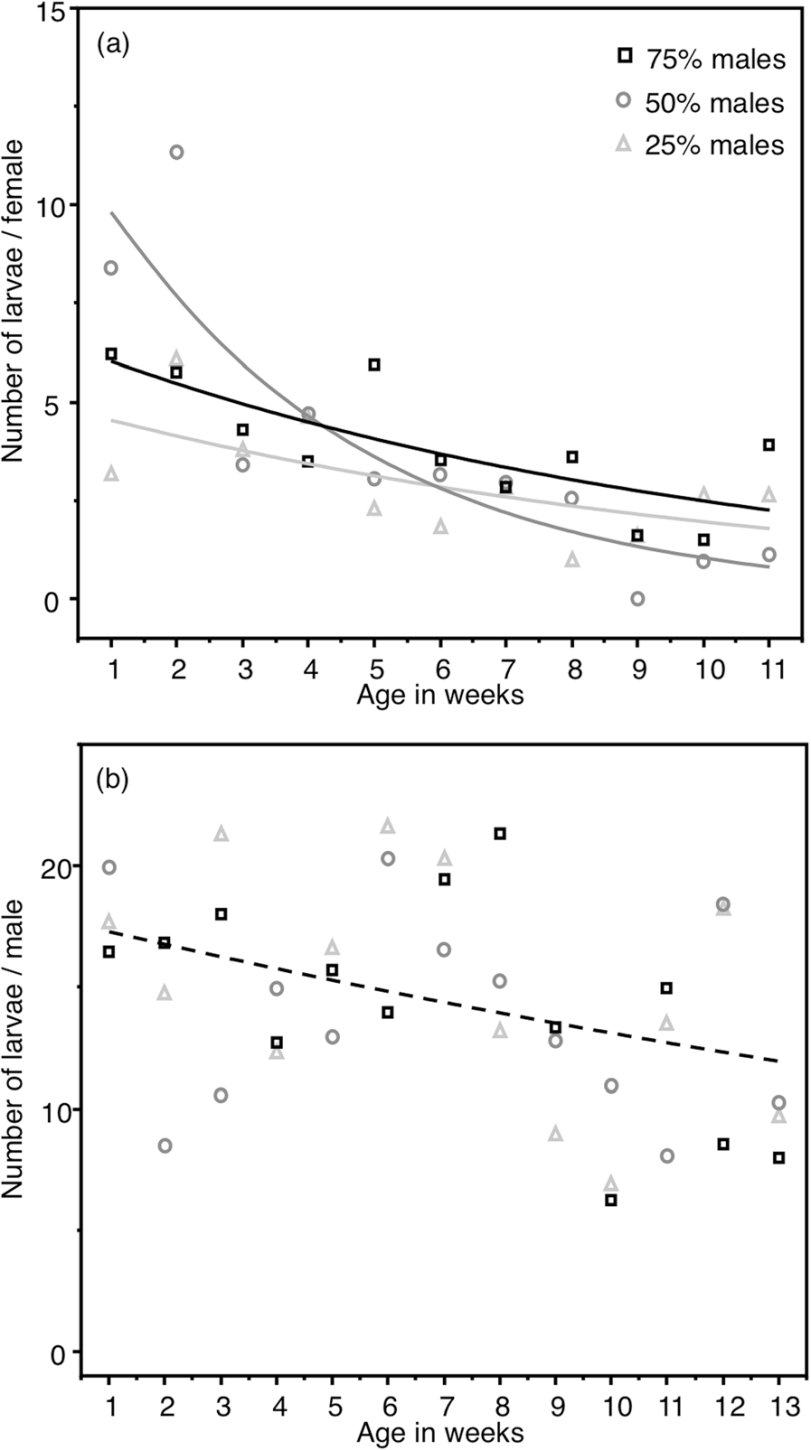
Age-specific fertility of females (**a**) and males (**b**) according to sex-ratio condition. **a** the tested females were those coming from the experimental tanks. **b** the tested females were virgin females mated with males coming from the experimental tanks. Dots are the means (for variation around the means see Additional file 1: Figure S2) and lines are the predictions of the models

Whereas female fertility decreased with time, this pattern was dependent on the sex-ratio condition (Table 2a, Fig. 2a, see Additional file 1: Figure S2). Indeed, female fertility in the 75 and 25%-male sex-ratio conditions was lower than that in the 50%-male sex-ratio condition during the first 2 weeks, and became subsequently higher (Fig. 2a). Male fertility decreased with time in all sex-ratio conditions, with no significant effect of sex-ratio condition (Table 2b, Fig. 2b, see Additional file 1: Figure S2). As expected for both sexes, heavier females produced more larvae than lighter ones (Table 2).

**Table 2:** Fertility: generalized linear mixt models (GLMM, Poisson distribution, Log link function) analysing the factors influencing the number of larvae produced by females (a), (*n* = 638) and males (b), (*n* = 737)

A				B			
Sources	*d.f.*	*χ2*	*p*	Sources	*d.f.*	*χ2*	*p*
Female mass	1	82.755	**< 0.001**	Female mass	1	243.13	**< 0.001**
Age	1	388.9	**< 0.001**	Age	1	94.819	**< 0.001**
Sex-ratio	2	0.7232	0.277				
Sex-ratio x Age	2	100.44	**< 0.001**				

Initial models included Sex-ratio condition, Age (in weeks as a continuous variable), their interaction, male and female body mass (Mass in mg) and replicates as a random factor. The models shown here are those minimizing the AICc, with δAICc > 2. Age was allowed to vary up to 11 and 13 weeks in females and males, respectively. Beyond these values no insects could be assayed anymore in some sex-ratio conditions (see Additional file 1: Figure S2). Values where *p* ≤ 0.05 are given in bold

**Fig. 3:**
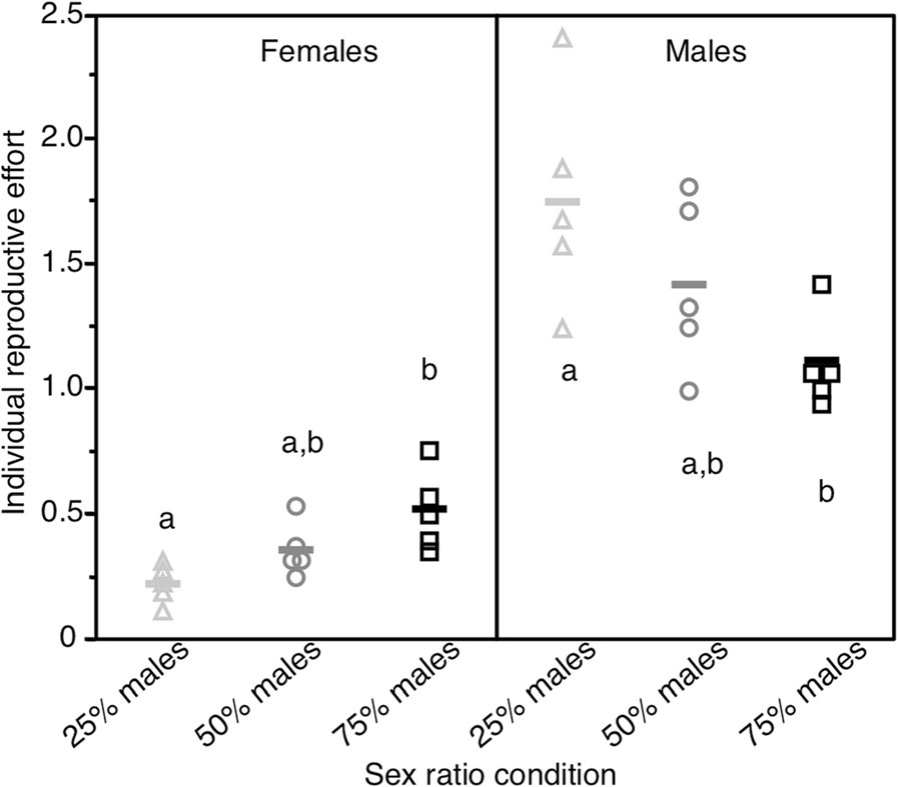
Estimated mean reproductive effort. Reproductive effort (RE - mean number of viable offspring produced per individual and per week of survival in the population) of females (left panel) and males (right panel) according to sex-ratio condition. Lines are means, dots are values of single replicates. Values surrounded by different letters were significantly different after Tukey-Kramer HSD post-hoc test (α = 0.05)

Female’s RE differed among sex-ratio conditions (*F*_2, 12_ = 8.06 *p* = 0.006) and was significantly higher in the 75%-male than in the 25%-male sex-ratio condition (Fig. 3a). Female RE in the 50%-male sex-ratio condition showed an intermediate value (Fig. 3a). Male RE significantly differed among sex-ratio conditions (*F*_2, 12_ = 4.63 *p* = 0.032). Male RE in the 75%-male sex-ratio condition was significantly lower than in the 25%-male sex-ratio condition (Fig. 3b). Like for females, male’s RE from the balanced sex-ratio condition showed an intermediate value, which was not significantly different from the two other sex-ratio conditions (Fig. 3b).

### Body condition and immunity

**Fig. 4:**
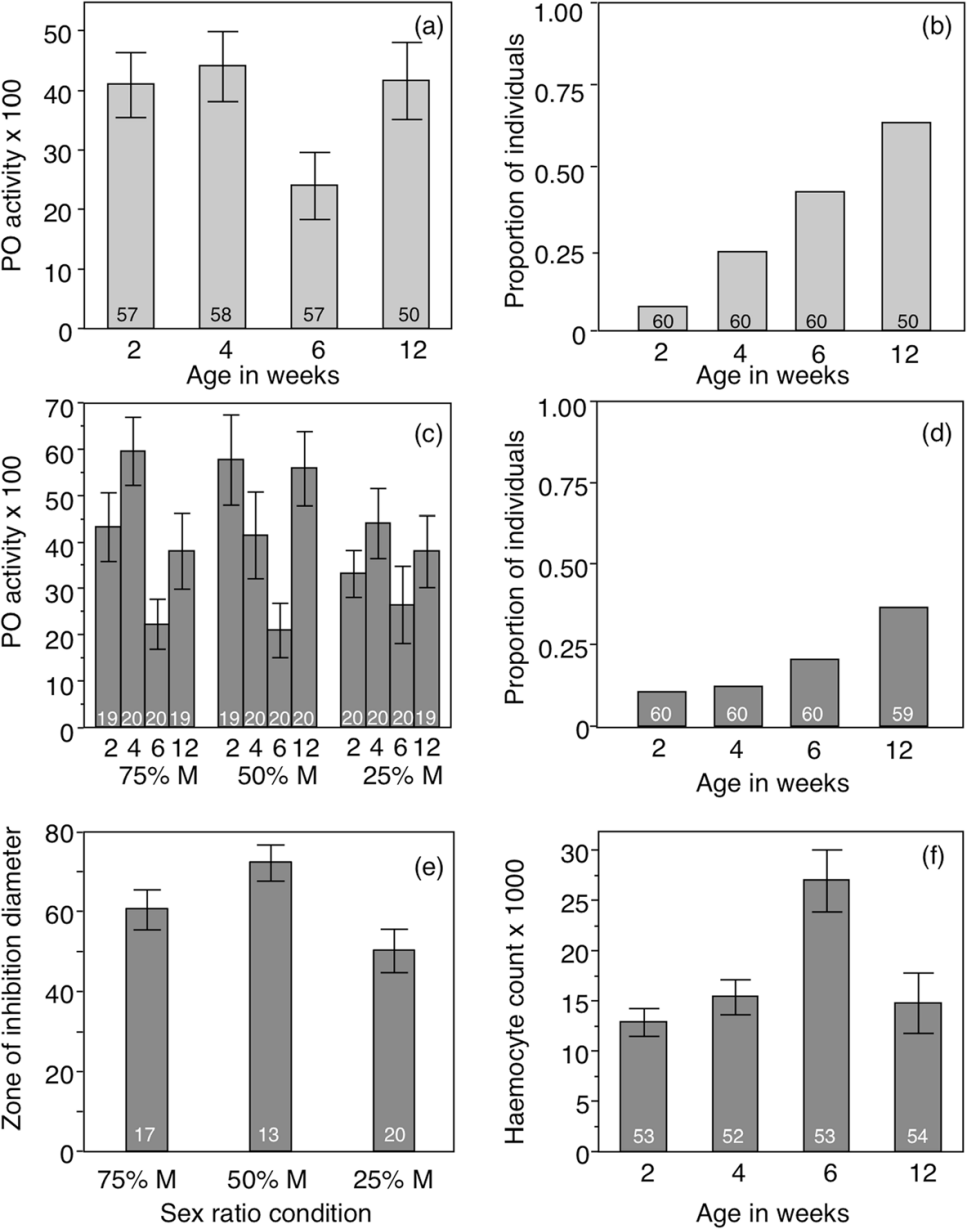
Immune parameters in females and males according to individual age and/or sex-ratio condition. **a** females PO activity; **b** proportion of females exhibiting antibacterial activity; **c** male PO activity; **d** proportion of males exhibiting antibacterial activity; **e** male intensity of antibacterial activity as the size (in mm) of zones of inhibition; **f** male haemocyte count. Values are means among replicates ± s. e. m. Number in the bars are sample size

Male and female body condition, estimated by the residuals of the regression between body size and body mass, exhibited a similar decline with age, which was not affected by the sex-ratio condition (Table 3a, see Additional file 1: Figure S3). In females, immunological parameters were never affected by the sex-ratio condition (Table 3b-f). Both PO activity and Total-PO activity changed with age with lowest values at week 6 (Fig. 4a), and were positively influenced by body condition (Table 3b, c). By contrast, anti-bacterial activity of the haemolymph increased with age (Table 3e, Fig. 4b). Haemocyte counts of females only differed among population replicates (Table 3d, see Additional file 1: Figure S3). As opposed to females, some of the immunological parameters of males were affected by the sex-ratio condition (Table 3). Male PO activity was influenced by the interaction between time and sex-ratio (Table 3b). While PO activity of males in the 50%-male sex-ratio condition decreased during the first 6 weeks, PO activity of males in the other two sex-ratio conditions increased between week 2 and 4. In all sex-ratio conditions, PO activity increased again at week 12 (Fig. 4c). In addition, PO activity of males in the 25%-male sex-ratio condition was overall lower than PO activity of males in the other sex-ratio conditions (Fig. 4c). Total-PO activity only differed between population replicates (Table 3c). As in females, antibacterial activity in the haemolymph of males increased with age (Table 3e, Fig. 4d). However, the size of the zones of inhibition of males exhibiting positive antibacterial activity (reflecting the intensity of this activity) was higher for males in the 50%-male sex-ratio condition than for males in the other sex-ratio conditions (Table 3f, Fig. 4e). Finally, haemocyte counts of males in all sex-ratio conditions varied with time, mainly because of its high value at week 6 (Table 3d, Fig. 4f).

**Table 3:** Body condition and immune parameters. Mixed linear models or generalized linear model analysing the factors influencing body condition (a), PO activity (b), Total PO activity (c), haemocyte count (d), the proportion of beetles exhibiting antibacterial activity in their haemolymph (e) and the intensity of this antibacterial activity as the size of the zone of inhibition (f) in both females (left) and males (right). Models included sex-ratio condition, Age in weeks (ordinal variable), their interaction, body condition, and replicates as a random factor

	Females				Males			
	*d.f.*	*d.f. den.*	*F or χ2*	*P*	*d.f.*	*d.f. den.*	*F or χ2*	*P*
A Body condition#								
Age	3	207.9	17.14	**< 0.001**	3	215.1	6.64	**< 0.001**
Sex-ratio	2	98.2	0.95	0.39	2	89.7	0.57	0.565
Age x Sex-ratio	6	207.6	0.72	0.637	6	215.1	0.23	0.964
B PO activity#								
Age	3	199.8	6.76	**< 0.001**	3	211.9	8.20	**< 0.001**
Sex-ratio	2	86.9	0.14	0.869	2	103.3	3.69	**0.028**
Age x Sex-ratio	6	198.3	0.75	0.606	6	211.3	2.21	**0.043**
Body condition	1	208.9	6.82	**< 0.001**	1	222.9	2.25	0.135
C Total-PO activity#								
Age	3	189	10.90	**< 0.001**	3	201.8	2.49	0.061
Sex-ratio	2	189	0.21	0.807	2	65.7	1.91	0.155
Age x Sex-ratio	6	189	0.40	0.877	6	201.2	1.25	0.282
Body condition	1	189	6.10	**0.014**	1	210.8	0.83	0.364
D Haemocyte#								
Age	3	193.8	1.28	0.281	3	208	6.90	**< 0.001**
Sex ratio	2	11.4	0.04	0.963				
E Antibacterial activity (proportion)§								
Age	3		42.89	**< 0.001**	3		12.879	**< 0.001**
Body condition	1		1.1902	0.275	1		10.595	0.157
Age					3		14.391	**0.002**
Sex-ratio					2		2.2331	0.327
F Antibacterial activity (intensity)#^1^								
Age	3		2.27	0.088				
Sex-ratio					2		4.27	**0.019**

The models presented here are those minimizing the AICc, with ΔAICc > 2. Values where *p* ≤ 0.05 are given in bold. # Linear model, § GLM, distribution: binomial, link function: Logit, ^1^ because a low number of animals showed antimicrobial activity in some conditions (Fig. 4b, d), data were not available for some replicates, therefore replicates were omitted for these models

## Discussion

By manipulating the sex-ratio of artificial populations of mealworm beetles, *Tenebrio molitor*, we successfully affected the reproductive effort of both males and females. Note that for males, our estimations are rather relevant of their reproductive potential effort or maximal reproductive effort because they were tested using young virgin females. As predicted, female biased sex-ratio led females to exhibit a relatively low reproductive effort, whereas males reproductive potential was the highest. By contrast, male biased sex-ratio increased the reproductive effort of females while that of males dropped. Males and females from populations with balanced sex-ratio exhibited intermediate reproductive effort. Interestingly, males and females of the 50% sex-ratio condition had similar longevity. This absence of divergent patterns of actuarial senescence between males and females may result from a relatively strong investment of males into mating activity rather than into sexual competition. Therefore, like in females, longevity appears to be an important criterion to maximize fitness in males of *T. molitor*.

While varying in their reproductive effort according to sex-ratio condition, females exhibited different patterns of actuarial and reproductive senescence. Females in the 75%-male sex-ratio condition (with the higher reproductive effort) suffered from an increased mortality compared to females in the other conditions. While we did not directly observed the mating behaviour in our experiments, frequent mating events and harassments by males may explain this accelerated mortality and is in line with previous observations from other insect species [34, 63–65].

Female fertility with time in the 50% sex-ratio condition contrasted to that of females in the other two sex-ratio conditions. They produced many offspring during the first 2 weeks of their adult life, then fewer to become almost null at 8 weeks onward. As previously reported in both vertebrates and invertebrates [22, 66], intense early reproductive activity is associated to earlier reproductive decline. Females in the other sex-ratio conditions (25 and 75% of males) produced relatively fewer offspring when young adults but kept this reproductive effort when becoming older. In the 25% sex-ratio condition, the low proportion of males may have constrained female access to mating, preventing them to reach their maximal early reproductive potential. Such a low early reproductive investment, possibly accompanied by low male harassment, may have preserved female late reproduction. In the 75% male sex-ratio condition, high proportion of males was expected to increase interactions between males as well as enhancing mate guarding [38]. This may also have prevented young females to have optimal access to mating, while compensating by a higher probability of mating as they aged. However, female reproduction in this male-biased sex-ratio condition stopped earlier than in the others, because their survival had rapidly declined. All together, the data suggest that the reproductive effort of females in the 75% male sex-ratio condition was more costly than that of females in the other sex-ratio conditions, constraining them to trade-off their longevity against their reproduction.

Despite a more costly reproductive effort, females in the 75%-male sex-ratio condition did not exhibit any further functional decline compare to females in the other sex-ratio conditions. While body condition declined with female age, such a decline was similar in all sex-ratio conditions. Similarly, changes in female immune activity were never influenced by the sex-ratio. While haemocyte counts remained constant with female age, antibacterial activity increased. Similar results were reported in the bumblebee, *Bombus terrestris* [53]. With age, the probability of having been exposed to microbes increases. This may explain the higher proportion of older individuals exhibiting induced antibacterial activity in their haemolymph, as insects can produce prophylactic long lasting antibacterial responses after a single bacterial challenge [67, 68]. PO activity declined at week 6 in females, which is consistent with the beginning of senescence, when reproduction started to end but survival is still relatively high (Fig. 1, see Additional file 1: Figure S2). PO activity increased again at week 12, among the rare surviving individuals (Fig. 1). This higher late PO-activity may have two non-exclusive explanations. On the one hand, it may result from selection where individuals with the best somatic protection, involving high PO-activity, survived longer than those having poorer ones. On the other hand, high levels of PO activity at week 12 could also result from a deregulation of the host inflammatory response [69, 70]. The impact of female reproductive effort on immunity seems limited, at least on the constitutive base levels of the immune parameters we have measured. We cannot exclude that female ability to produce an immune response upon challenge or others non-measured immune parameters could be affected. Nonetheless, our results show that increasing the reproductive effort of *T. molitor* females affected demographic senescence, mainly through longevity reduction, but with apparently limited effect on immune senescence.

Changes in the reproductive effort (reproductive potential) of males through the manipulation of the sex-ratio also affected their survival. It is often assumed that most of the cost of reproduction in males involves sexual pre-copulatory competition. Thus males in the 75%-male sex-ratio condition could have been expected to engage in strong and costly intra-sexual competition for females, resulting in low reproductive success and shorter longevity compared to the other sex-ratio conditions, as previously shown in vertebrates [11, 71, 72] and invertebrates [34]. However, although male fertility slightly declined with age, it was not affected by the experimental sex-ratio condition, suggesting that males exhibited similar patterns of reproductive senescence, independently of their reproductive effort. Male reproductive senescence might also be revealed by the production of lower quality offspring with age [73], which was not tested in this study. In addition, males from the 75%-sex-ratio condition showed longer longevity. This phenomenon may have two main explanations, consistent with predictions linked to the peculiar mating behaviour of *T. molitor*. On the one hand, competition for females in that sex-ratio condition was not very strong or costly. Under high risk of sexual competition, male reproductive success may depend on their investment into pre-copulatory (e.g., courtship and aggressive behaviours with other males) and/or post-copulatory (e.g., mate guarding) behaviours to limit sperm competition [74]. So far, *T. molitor* males were never reported to engage in physical contest either before or after copulation [38] and current evidence suggests that male-male competition is unlikely to bear strong costs [38, 41, 75, 76]. Our experimental design nevertheless did not allowed us to verify these assumptions. On the other hand, each mating event is costly for *T. molitor* males, because nutrient-rich spermatophores are transferred to females in addition to the sperm [42]. Since, on average, males in the 75%-male sex-ratio condition may have copulated less frequently than males in the other sex-ratio conditions, they may have saved resources that contributed to their longer survival. By contrast, males from the 25%-male sex-ratio condition likely performed more mating events than males in the other sex-ratio conditions, but this was apparently not costly enough to significantly impair their longevity compared to the 50%-male sex-ratio condition.

Our results suggest that males in this 25%-male sex-ratio condition had paid a functional cost for their higher reproductive effort, especially in terms of immunity. Indeed, males in the 25%-male sex-ratio condition showed a reduced immune activity possibly resulting from their higher reproductive effort. First, males in the 25%-male sex-ratio condition had reduced PO activity despite having a similar concentration of total phenoloxidase enzymes in their haemolymph than males of the other sex-ratio conditions. This lower PO activity was constant over time and contrasted with that of males of the two other sex-ratio conditions, for which the temporal pattern of PO activity resembled that of females (high levels in early weeks, decline in week 6, and re-increase in week 12). Since mating activity is known to transiently reduce PO activity in *T. molitor* [52], such a down regulation of the PO activity in males of the 25%-male sex-ratio condition might be reflecting their higher mating activities. Higher secretion of juvenile hormone might be involved in mediating mating-induced PO activity depression [52], which could contribute to reduce longevity [77]. Juvenile hormone also prevents the release of cytotoxic substances by active PO enzymes that could reduce insect longevity by self-damaging host tissues and organs [47, 49–51, 78]. These combined effects may have contributed to the observed absence of difference between the survival of males in the 25% and the 50%-male sex-ratio conditions. Second, as observed for females, the proportion of males exhibiting positive antibacterial activity in their haemolymph increased with age in all the sex-ratio conditions. As stated earlier, this was expected as the probability of having being challenged by opportunistic microbes increases with age. However, the size of the zones of inhibition observed from the haemolymph of males in the 25%-male sex-ratio condition was significantly smaller than that of males in the other sex-ratio conditions, suggesting that males in the 25%-male sex-ratio condition produced less antibacterial peptides than the other males. As mating activity, through the production and transfer of spermatophores, is particularly resource-demanding for males in terms of protein content [42], the higher mating activity of males in the 25%-male sex-ratio condition could have depleted the necessary protein resource to produce as much antibacterial peptides as in the other sex-ratio conditions. Mating may mediate such a trade-off through juvenile hormone secretion, which functions to switch on physiological processes associated with gametogenesis and spermatophore production [79].

## Conclusions

Manipulating sex-ratio of artificial populations of *T. molitor* had important impacts on reproductive effort of females and males, but resulted in contrasting sex-specific trade-offs on demographic and immune traits. Increasing female reproductive effort did not affect immunity but strongly reduced longevity. Not surprisingly, females may then maximize fitness by moderate early investment into reproduction and longevity. While decreasing male reproductive effort enhanced longevity, increasing it impairs immunity. Males may therefore favour reproduction at the expense of their immunity when given the opportunity to increase their reproductive effort. This is in line with the Bateman’s principle applied to immunity, where males gain fitness by increasing reproductive effort at the expense of immunity [80]. It is also consistent with the disposable soma theory of ageing, as reproduction compromises somatic protection [3, 4]. Nevertheless, our results also suggest that sexual competition in *T. molitor* is not a strong modulator of the male reproductive strategy towards early mating opportunities [81]. Basically, like in females, most of the cost of reproduction in males results from multiple copulations. This thus contrasts with the hypothesis that males should gain fitness by increasing mating success by investing in sexual competition at the expense of longevity [82, 83]. Since longevity is a key life history trait for both males and females of *T. molitor*, sex-specific patterns of actuarial senescence are not expected to evolve in this species. Accordingly, males and females showed similar patterns of survival with age in populations with balanced sex-ratio. Our results may further indirectly suggest that divergent actuarial senescence between males and females should evolve in species in which males strongly invest into sexual competition [11, 22].

## Supplementary information

**Additional file 1: Table S1.** AICc values for the models presented in Tables 2 and 3. The models in bold are those presented in the tables. **Figure S1.** Male and female age specific mortality rate according to time for each sex-ratio condition. Arrow indicate the time when the values of 50% of mortality rate is reached for the first time and dashed lines indicate 50% of the population is dead. Values are means among replicates ± s. e. m. **Figure S2.** Details of variation in fertility in females (A) and males (B). Values are means among replicates ± s. e. m. **Figure S3.** Physiological parameters: females in bright grey and males in black. Body condition, PO activity according to time, Total-PO activity according to time and sex-ratio condition, Haemocyte count, Proportion of individuals producing antibacterial activity, Diameter of inhibition zone according to time and sex-ratio condition. Values are means among replicates ± s. e. m.

## Data Availability

All data files will be available from the Dryad Digital database at 10.5061/dryad.cvdncjt11.

## Abbreviations

AICc: Corrected Akaike Information Criterion
ANOVA: Analysis of Variance
*l*: Number of larvae
ML: Mean Lifespan
PBS: Phosphate Buffer Saline
PO: Phenoloxidase
ProPO: ProPhenoloxidase
*r*: Replicate
RE: Reproductive Effort
REML: REstricted Maximum Likelihood
