# Supplementary material for: Sex-specific patterns of senescence in artificial insect populations varying in sex-ratio to manipulate reproductive effort
Source: BMC Evol Biol. 2020 Feb 3;20:18. doi: 10.1186/s12862-020-1586-x (PMC6998128; doi:10.1186/s12862-020-1586-x)
Supplement: Supplementary file 1 — Additional file 1: Table S1. AICc values for the models presented in Tables 2 and 3. The models in bold are those presented in the tables. Figure S1. Male and female age specific mortality rate according to time for each sex-ratio condition. Arrow indicate the time when the values of 50% of mortality rate is reached for the first time and dashed lines indicate 50% of the population is dead. Values are means among replicates ± s. e. m. Figure S2. Details of variation in fertility in females (A) and males (B). Values are means among replicates ± s. e. m. Figure S3. Physiological parameters: females in bright grey and males in black. Body condition, PO activity according to time, Total-PO activity according to time and sex-ratio condition, Haemocyte count, Proportion of individuals producing antibacterial activity, Diameter of inhibition zone according to time and sex-ratio condition. Values are means among replicates ± s. e. m. [file 12862_2020_1586_MOESM1_ESM.docx]

**Additional file 1**

Sex-specific patterns of senescence in artificial insect populations varying in sex-ratio to manipulate reproductive effort

**Table S1:** AICc values for the models presented in Tables 2 and 3. The models in bold are those presented in the tables.

Fertility females

| **Models** | **AICc** |
| --- | --- |
| **Fertility ~ Age * Sex-ratio + Female Mass + (\|replicate)** | **7627.5** |
| Fertility ~ Age + Female Mass + (\|replicate) | 7668.2 |
| Fertility ~ Age + Sex-ratio + Female Mass + (\|replicate) | 7671.5 |
| Fertility ~ Age * Sex-ratio + (\|replicate) | 7773.8 |
| Fertility ~ Age + (\|replicate) | 7777.3 |
| Fertility ~ Age + Sex-ratio + (\|replicate) | 7779.0 |
| Fertility ~ Female Mass + (\|replicate) | 8089.8 |
| Fertility ~ Sex-ratio + Female Mass + (\|replicate) | 8092.2 |
| Fertility ~ Sex-ratio + (\|replicate) | 8325.3 |

Fertility males

| **Models** | **AICc** |
| --- | --- |
| **Fertility ~ Age + Female Mass + (\|replicate)** | **16522.1** |
| Fertility ~ Age * Sex-ratio + Female Mass + Male Mass + (\|replicate) | 16530.8 |
| Fertility ~ Age + Sex-ratio + Female Mass + (\|replicate) | 16532.9 |
| Fertility ~ Age + Female Mass + Male Mass + (\|replicate) | 16533.7 |
| Fertility ~ Age + Sex-ratio + Female Mass + Male Mass + (\|replicate) | 16534.4 |
| Fertility ~ Female Mass + Male Mass + (\|replicate) | 16725.5 |
| Fertility ~ Sex-ratio + Female Mass + Male Mass + (\|replicate) | 16725.8 |
| Fertility ~ Female Mass + (\|replicate) | 16730.5 |
| Fertility ~ Sex-ratio + Female Mass + (\|replicate) | 16731.2 |
| Fertility ~ Age + Sex-ratio + (\|replicate) | 16778.7 |
| Fertility ~ Age + (\|replicate) | 16779.0 |
| Fertility ~ Age + Sex-ratio + Male Mass + (\|replicate) | 16780.7 |
| Fertility ~ Age + Male Mass + (\|replicate) | 16781.0 |
| Fertility ~ Sex-ratio + Male Mass + (\|replicate) | 17057.5 |
| Fertility ~ Male Mass + (\|replicate) | 17058.3 |
| Fertility ~ Sex-ratio + (\|replicate) | 17060.5 |

Body condition females

| **Models** | **AICc** |
| --- | --- |
| **Body condition ~ Age + Sex ratio + Age*Sex-ratio + (\|replicate)** | **1700.5** |
| Body condition ~ Age + Sex ratio + (\|replicate) | 1712.1 |
| Body condition ~ Age + (\|replicate) | 1714.6 |
| Body condition ~ Sex ratio + (\|replicate) | 1760.6 |

Body condition males

| **Models** | **AICc** |
| --- | --- |
| **Body condition ~ Age + Sex ratio + Age*Sex-ratio + (\|replicate)** | **1785.8** |
| Body condition ~ Age + Sex ratio + (\|replicate) | 1794.5 |
| Body condition ~ Age + (\|replicate) | 1794.7 |
| Body condition ~ Sex ratio + (\|replicate) | 1816.5 |

PO females

| **Models** | **AICc** |
| --- | --- |
| **PO ~ Age + Sex ratio + Age*Sex-ratio + Body condition + (\|replicate)** | **2164.3** |
| PO ~ Age + Sex ratio + Age*Sex-ratio + (\|replicate) | 2167.8 |
| PO ~ Age + Sex ratio + Body condition + (\|replicate) | 2191.0 |
| PO ~ Age + Sex ratio + (\|replicate) | 2194.2 |
| PO ~ Age + Body condition + (\|replicate) | 2195.9 |
| PO ~ Age + (\|replicate) | 2198.4 |
| PO ~ Sex ratio + (\|replicate) | 2219.5 |
| PO ~ Sex ratio + Body condition + (\|replicate) | 2220.1 |
| PO ~ Body condition + (\|replicate) | 2224.9 |

PO males

| **Models** | **AICc** |
| --- | --- |
| **PO ~ Age + Sex ratio + Age*Sex-ratio + (\|replicate)** | **2318.2** |
| **PO ~ Age + Sex ratio + Age*Sex-ratio + Body condition + (\|replicate)** | **2319.3** |
| PO ~ Age + Sex ratio + (\|replicate) | 2353.9 |
| PO ~ Age + Sex ratio + Body condition + (\|replicate) | 2354.6 |
| PO ~ Age + (\|replicate) | 2362.2 |
| PO ~ Age + Body condition + (\|replicate) | 2362.9 |
| PO ~ Sex ratio + (\|replicate) | 2386.3 |
| PO ~ Sex ratio + Body condition + (\|replicate) | 2387.5 |
| PO ~ Body condition + (\|replicate) | 2395.9 |

Total PO females

| **Models** | **AICc** |
| --- | --- |
| **Total PO ~ Age + Sex ratio + Age*Sex-ratio + Body condition + (\|replicate)** | **2044.4** |
| **Total PO ~ Age + Sex ratio + Age*Sex-ratio + (\|replicate)** | 2047.7 |
| Total PO ~ Age + Sex ratio + Body condition + (\|replicate) | 2071.9 |
| Total PO ~ Age + Sex ratio + (\|replicate) | 2075.0 |
| Total PO ~ Age + Body condition + (\|replicate) | 2079.2 |
| Total PO ~ Age + (\|replicate) | 2081.8 |
| Total PO ~ Sex ratio + (\|replicate) | 2112.4 |
| Total PO ~ Sex ratio + Body condition + (\|replicate) | 2114.3 |
| Total PO ~ Body condition + (\|replicate) | 2122.0 |

Total PO males

| **Models** | **AICc** |
| --- | --- |
| **Total PO ~ Age + Sex ratio + Age*Sex-ratio + (\|replicate)** | **2429.1** |
| **Total PO ~ Age + Sex ratio + Age*Sex-ratio + Body condition + (\|replicate)** | **2430.6** |
| Total PO ~ Age + Sex ratio + (\|replicate) | 2465.6 |
| Total PO ~ Age + Sex ratio + Body condition + (\|replicate) | 2466.7 |
| Total PO ~ Age + (\|replicate) | 2475.3 |
| Total PO ~ Age + Body condition + (\|replicate) | 2476.5 |
| Total PO ~ Sex ratio + (\|replicate) | 2485.5 |
| Total PO ~ Sex ratio + Body condition + (\|replicate) | 2487.2 |
| Total PO ~ Body condition + (\|replicate) | 2496.9 |

Haemocytes females

| **Models** | **AICc** |
| --- | --- |
| **Haemocytes ~ Age + (\|replicate)** | **573.4** |
| **Haemocytes ~ Sex ratio + (\|replicate)** | **574.6** |
| Haemocytes ~ Body condition + (\|replicate) | 575.6 |
| Haemocytes ~ Age + Sex ratio + (\|replicate) | 582.8 |
| Haemocytes ~ Age + Body condition + (\|replicate) | 583.9 |
| Haemocytes ~ Sex ratio + Body condition + (\|replicate) | 584.8 |
| Haemocytes ~ Age + Sex ratio + Body condition + (\|replicate) | 593.3 |
| Haemocytes ~ Age + Sex ratio + Age*Sex-ratio + (\|replicate) | 598.9 |
| Haemocytes ~ Age + Sex ratio + Age*Sex-ratio + Body condition + (\|replicate) | 609.5 |

Haemocytes males

| **Models** | **AICc** |
| --- | --- |
| **Haemocytes ~ Age + (\|replicate)** | **608.7** |
| Haemocytes ~ Age + Body condition + (\|replicate) | 618.0 |
| Haemocytes ~ Age + Sex ratio + (\|replicate) | 618.4 |
| Haemocytes ~ Body condition + (\|replicate) | 626.1 |
| Haemocytes ~ Sex ratio + (\|replicate) | 626.8 |
| Haemocytes ~ Age + Sex ratio + Body condition + (\|replicate) | 627.8 |
| Haemocytes ~ Sex ratio + Body condition + (\|replicate) | 635.8 |
| Haemocytes ~ Age + Sex ratio + Age*Sex-ratio + (\|replicate) | 637.4 |
| Haemocytes ~ Age + Sex ratio + Age*Sex-ratio + Body condition + (\|replicate) | 646.8 |

Antibacterial activity (proportion) females

| **Models** | **AICc** |
| --- | --- |
| **Antibacterial activity ~ Age (\|replicate)** | **259.4** |
| **Antibacterial activity ~ Age + Body condition + (\|replicate)** | **260.2** |
| Antibacterial activity ~ Age + Sex ratio + (\|replicate) | 262.3 |
| Antibacterial activity ~ Age + Sex ratio + Body condition + (\|replicate) | 262.9 |
| Antibacterial activity ~ Age + Sex ratio + Age*Sex-ratio +(\|replicate) | 266.1 |
| Antibacterial activity ~ Age + Sex ratio + Age*Sex-ratio + Body condition +(\|replicate) | 266.2 |
| Antibacterial activity ~ Body condition + (\|replicate) | 297.1 |
| Antibacterial activity ~ Sex ratio (\|replicate) | 300.2 |
| Antibacterial activity ~ Sex ratio + Body condition + (\|replicate) | 300.7 |

Antibacterial activity (proportion) males

| **Models** | **AICc** |
| --- | --- |
| **Antibacterial activity ~ Age (\|replicate)** | **241** |
| **Antibacterial activity ~ Age + Sex ratio + (\|replicate)** | **242.8** |
| **Antibacterial activity ~ Age + Body condition + (\|replicate)** | **242.8** |
| Antibacterial activity ~ Age + Sex ratio + Age*Sex-ratio +(\|replicate) | 244.4 |
| Antibacterial activity ~ Age + Sex ratio + Body condition + (\|replicate) | 244.6 |
| Antibacterial activity ~ Age + Sex ratio + Age*Sex-ratio + Body condition +(\|replicate) | 246.2 |
| Antibacterial activity ~ Body condition + (\|replicate) | 249.9 |
| Antibacterial activity ~ Sex ratio (\|replicate) | 250.8 |
| Antibacterial activity ~ Sex ratio + Body condition + (\|replicate) | 251.8 |

Antibacterial activity (intensity) females

| **Models** | **AICc** |
| --- | --- |
| **Antibacterial activity ~ Age** | **676.3** |
| Antibacterial activity ~ Age + Body condition | 678.5 |
| Antibacterial activity ~ Body condition | 678.8 |
| Antibacterial activity ~ Sex ratio | 680.6 |
| Antibacterial activity ~ Age + Sex ratio | 681.1 |
| Antibacterial activity ~ Sex ratio + Body condition | 682.8 |
| Antibacterial activity ~ Age + Sex ratio + Body condition | 683.2 |
| Antibacterial activity ~ Age + Sex ratio + Age*Sex-ratio | 693.9 |
| Antibacterial activity ~ Age + Sex ratio + Age*Sex-ratio + Body condition | 695.3 |

Antibacterial activity (intensity) males

| **Models** | **AICc** |
| --- | --- |
| **Antibacterial activity ~ Sex ratio** | **452.8** |
| Antibacterial activity ~ Sex ratio + Body condition | 455.0 |
| Antibacterial activity ~ Body condition | 458.0 |
| Antibacterial activity ~ Age + Sex ratio | 458.3 |
| Antibacterial activity ~ Age | 460.2 |
| Antibacterial activity ~ Age + Sex ratio + Body condition | 461.1 |
| Antibacterial activity ~ Age + Body condition | 462.6 |
| Antibacterial activity ~ Age + Sex ratio + Age*Sex-ratio | 470.9 |
| Antibacterial activity ~ Age + Sex ratio + Age*Sex-ratio + Body condition | 474.5 |

| 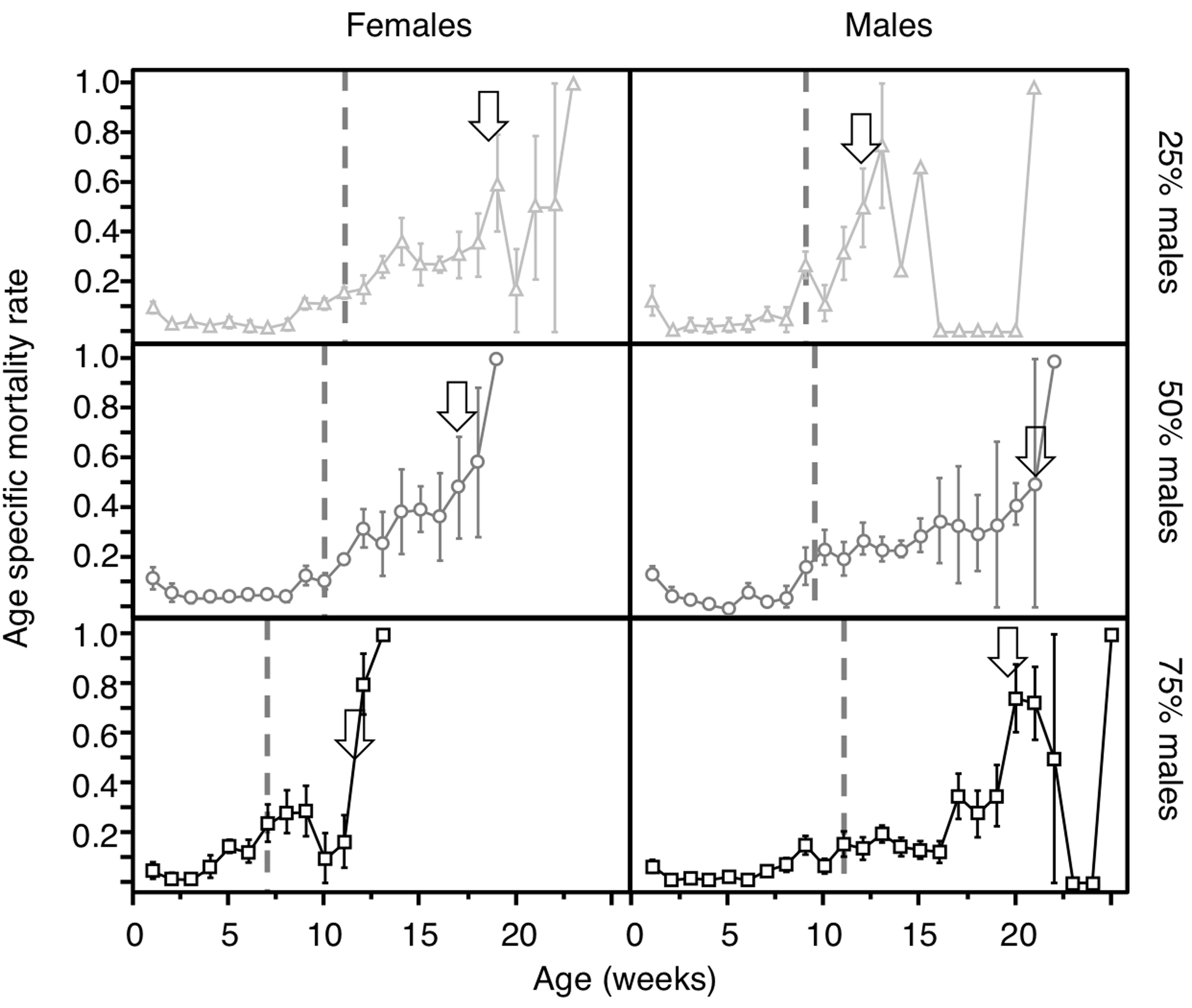 |
| --- |
| Figure. S1. Male and female age specific mortality rate according to time for each sex-ratio condition. Arrows indicate the time when the value of 50% of mortality rate is reached for the first time and dashed lines indicate 50% of the population is dead. Values are means among replicates ± s. e. m |

| 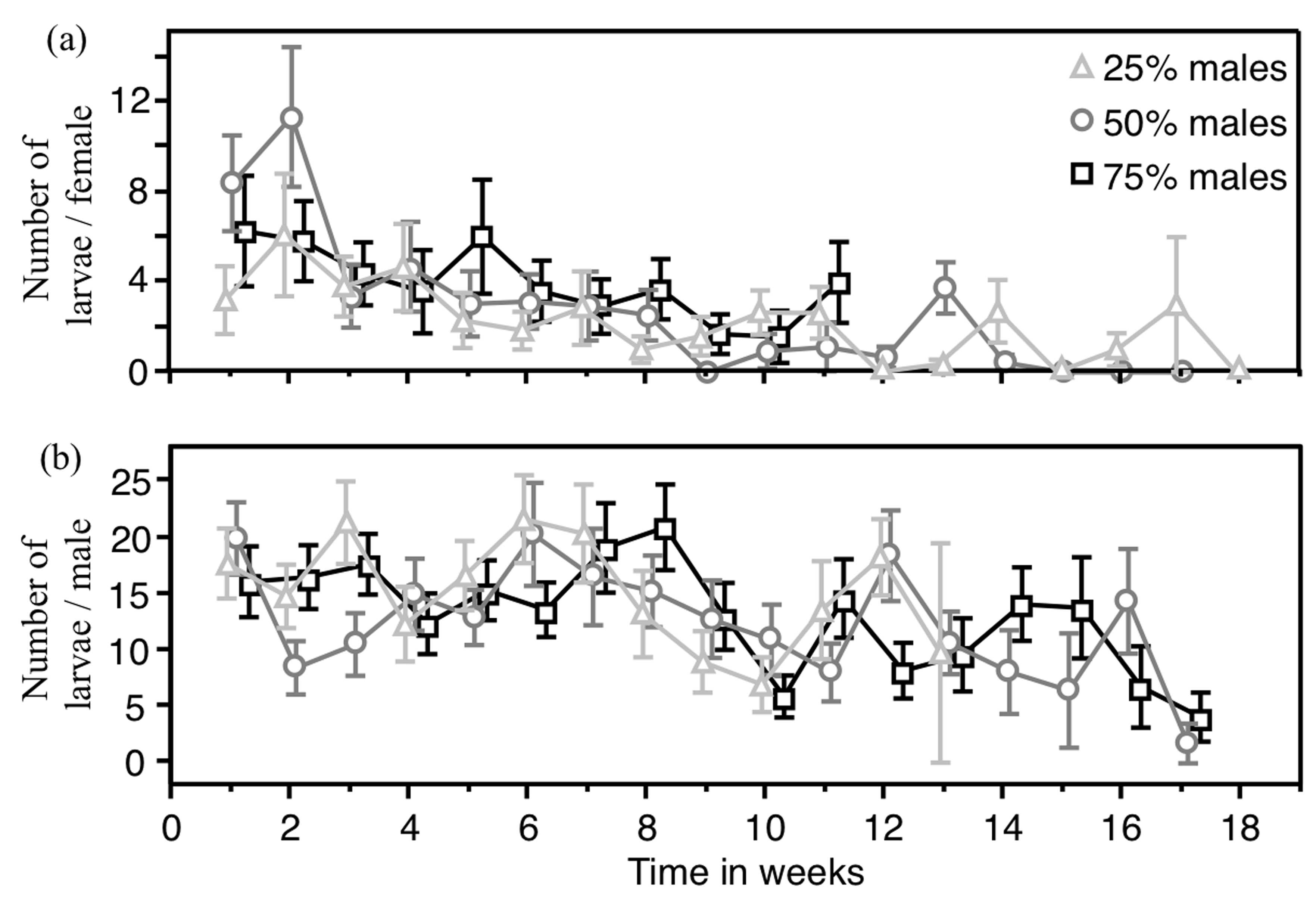 |
| --- |
| Figure. S2. Details of variation in fertility in females (A) and males (B). Values are means among replicates ± s. e. m. |

| 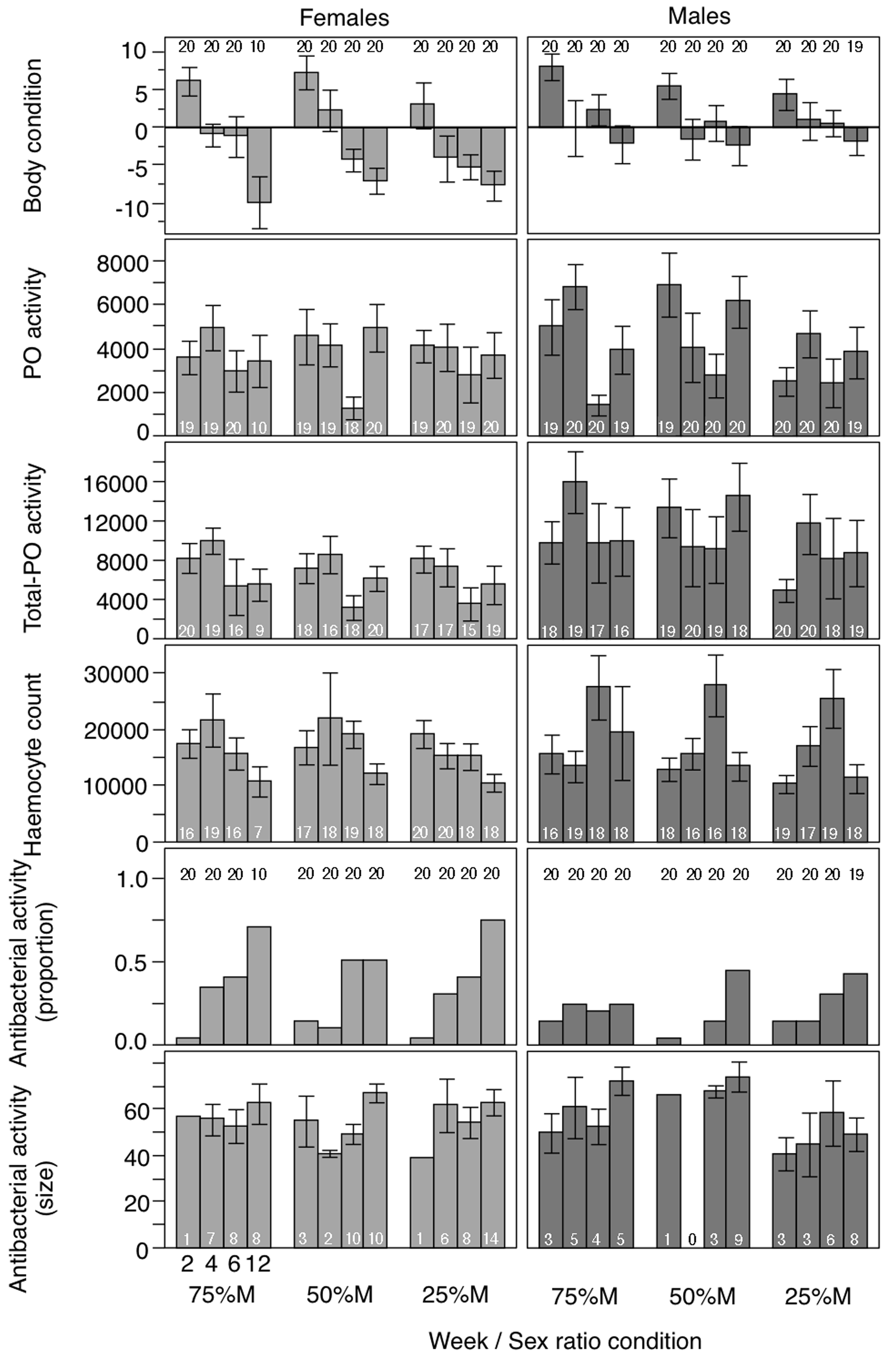 |
| --- |
| Figure. S3. Physiological parameters: females in bright grey and males in black. Body condition, PO activity according to time, Total-PO activity according to time and sex-ratio condition, Haemocyte count, Proportion of individuals producing antibacterial activity, diameter of inhibition zone according to time and sex-ratio condition. Values are means among replicates ± s. e. m. |
